# Exploring difference in atrioventricular valve opening times to predict elevated left atrial pressure - a novel approach to left atrial pressure quantification on cardiovascular MRI

**DOI:** 10.1186/1532-429X-18-S1-P192

**Published:** 2016-01-27

**Authors:** Mohan Mallikarjuna Rao Edupuganti, Bennett Battle, Shelly Lensing, Tarun Pandey

**Affiliations:** 1 grid.241054.60000000122929177Radiology, University of Arkansas for Medical Sciences, Little Rock, AR USA; 2 grid.241054.60000000122929177Cardiology, University of Arkansas for Medical Sciences, Little Rock, AR USA; 3 grid.241054.60000000122929177Biostatistics, University of Arkansas for Medical Sciences, Little Rock, AR USA

## Background: Purpose

To study if difference between the tricuspid and mitral valve opening time evaluated using cardiac magnetic resonance imaging (CMR) correlates with echocardiographic measurements of left atrial pressure (LAP).

## Background

The ratio of the transmitral early inflow velocity (E wave) and the tissue Doppler mitral annular velocity (e' wave) can be used to measure left atrial pressure on the echocardiographic examination. An elevated E/e' (>15) on echo Doppler is an established measure of elevated LAP.

Unlike echo, cardiac MRI (CMR) has no non-invasive measure of LAP. Normally both the atrioventricular valves open simultaneously or the tricuspid valve opens earlier than the mitral valve due to a very short right-sided isovolumic relaxation time (IVRT). Elevated LAP shortens the left sided IVRT and therefore causes the mitral valve to open earlier than expected. Also, elevated LAP indirectly leads to elevation of right heart pressures causing lengthening of the tricuspid valve isovolumic relaxation time resulting in delayed tricuspid valve opening. We investigate the role of using the time differential of mitral and tricuspid opening as novel parameter of LAP measurement on CMR.

## Hypothesis

We hypothesize that elevated LAP should lead to an increased positive time differential of the mitral and tricuspid valve opening times.

## Methods

CMR and echo Doppler studies in 32 patients were reviewed. Echo Doppler data were reviewed to measure the mitral E wave and septal e' velocities. The LAP was calculated using the following formula:

LAP = (E/e' x 1.25) + 1.9

12 out of 30 patients met the criteria for an elevated left atrial pressure based on the E/e' ratios and the remaining 20 patients had normal LAP.

On CMR, the mitral and tricuspid valve opening times (in milliseconds) were calculated using the 2D short axis cine phase contrast images obtained at the level of the AV valves. The velocity versus time graphs and cine images were used to calculate and differences in valve opening times.

The correlation between the CMR derived mitral and tricuspid valve time differential and echocardiographic E/e' derived LAP values was calculated using Spearman's rank correlation.

## Results

Our study showed that there is significant correlation between the echo derived LAP using E/e' and mitral and tricuspid valve opening time differential. Spearman's rank correlation was 0.39 (p=0.028). A positive time differential (mitral valve opening earlier than tricuspid valve) had high specificity in detecting elevated LAP (85%). However, the sensitivity was poor (50%). The positive and negative predictive values were 67% and 74%, respectively.

## Conclusions

Time difference between the openings of the atrioventricular valves is a highly specific marker of elevated left atrial pressure. This marker merits further testing to determine if it can provide quantitative information on the degree of left atrial pressure elevation.

